# The potential shared brain functional alterations between adults with ADHD and children with ADHD co-occurred with disruptive behaviors

**DOI:** 10.1186/s13034-022-00486-7

**Published:** 2022-06-27

**Authors:** Ningning Liu, Gaoding Jia, Haimei Li, Shiyu Zhang, Yufeng Wang, Haijing Niu, Lu Liu, Qiujin Qian

**Affiliations:** 1 Peking University Sixth Hospital/Institute of Mental Health, Beijing, 100191 China; 2 NHC Key Laboratory of Mental Health (Peking University), National Clinical Research Center for Mental Disorders (Peking University Sixth Hospital), Beijing, 100191 China; 3 State Key Laboratory of Cognitive Neuroscience and Learning, Beijing Normal University, Beijing, 100875 China

**Keywords:** ADHD Development, Disruptive behaviour disorders, fNIRS, Functional connectivities

## Abstract

**Background:**

Attention-deficit/hyperactivity disorder (ADHD) is a common neurodevelopmental disorder. Many previous studies have shown that the comorbid status of disruptive behaviour disorders (DBD) was a predictor for ADHD persistence into adulthood. However, the brain mechanisms underlying such a relationship remain unclear. Thus, we aim to investigate whether the brain functional alteration in adults with ADHD could also be detected in children with ADHD co-occurring with disruptive behaviours from both quantitative and categorical dimensions.

**Methods:**

A total of 172 children with ADHD (cADHD), 98 adults with ADHD (aADHD), 77 healthy control children (cHC) and 40 healthy control adults (aHC) were recruited. The whole-brain spontaneous fluctuations in brain activity of each participant were recorded using functional near-infrared spectroscopy (fNIRS), and the functional connectivities (FCs) were calculated. We first compared the FC differences between aADHD and aHC. Then, for the regions with significantly abnormal FCs in aADHD, we further compared these features between cADHD and cHC. In addition, the correlation between these FCs and the conduct disorder (CD)/oppositional defiant disorder (ODD) symptoms were analysed in cADHD. Moreover, to render the results readily interpretable, we compared the FC differences among ADHD_CD−_, subthreshold ADHD_CD+_ and cHC groups, and among ADHD_ODD−_, ADHD_ODD+_ and cHC groups. Finally, we repeated the above analysis after controlling for other comorbidities and core symptoms to diminish the potential confounding effects.

**Results:**

We found that compared with aHC, aADHD showed significantly increased FCs in the VN, DMN, SMN, and DAN. The aforementioned abnormal FCs were also detected in cADHD, however, in an opposite orientation. Notably, these abnormal FCs were positively correlated with CD symptoms. Finally, the subthreshold ADHD_CD+_ group even exhibited a tendency of adult-like increased FCs compared with the cHC. The results held after controlling for other comorbidities and core symptoms.

**Conclusion:**

This study provides functional neuroimaging evidence that CD might be a risk factor for ADHD persistence into adulthood. Our work highlights the importance of differentiating ADHD_CD+_ from ADHD and inspiring further understanding of brain development in ADHD.

**Supplementary Information:**

The online version contains supplementary material available at 10.1186/s13034-022-00486-7.

## Introduction

Attention-deficit/hyperactivity disorder (ADHD) is a common neurodevelopmental disorder associated with many adverse life events and creates a substantial burden for individuals, their families, and the community. The ADHD persistence rate is in the range of 5.7–77%, and there are many factors associated with the course of ADHD [1].

ADHD in children often co-occurs with many comorbidities, such as oppositional defiant disorder (ODD) and conduct disorder (CD), which are collectively referred to as disruptive behaviour disorders (DBD). DBD is a common and highly impairing psychiatric disorder characterized by conduct problems, irritability, and oppositional defiant behaviour [2]. Children with ADHD and DBD (ADHD_DBD+_) have additional impairments and worse prognosis than children with ADHD alone or DBD alone [3, 4]. In particular, many empirical studies have shown that comorbid DBD predicts ADHD persistence through adulthood. For instance, Biederman et al. found that adolescents and adults with persistent ADHD were more likely to have DBD problems in childhood than those with desistent ADHD [5]. Later, in girls with ADHD, the same group also found that the persistent ADHD group had significantly higher rates of DBD at baseline [6]. Similarly, Eric et al. found that DBD predicts ADHD persistence in girls at a 5-year follow-up study [7]. Another study also indicated that irritability, which is a common characteristic of DBD, might play a key role in the persistence and worsening of hyperactive/impulsive symptoms across adolescence for females [8]. Recently, one study found that comorbid DBD is one of the most consistently observed predictors of functional outcomes [1]. Despite much research, no consideration has been given to the underlying brain mechanisms for the phenomenon.

In recent decades, several neuroimaging studies have been conducted on individuals with ADHD and DBD, which have produced additional insight into the pathophysiological mechanisms of ADHD and DBD. Therefore, can these studies provide some hints and tips about the above-mentioned phenomenon? The answer is yes. For example, for structural morphology, many studies found more significantly or more extensively decreased fractional anisotropy (FA), white matter (WM) volume and grey matter (GM) volume when the effect of comorbid DBD was taken into account, including the basal ganglia, cerebellum, and frontal cortices [9, 10]. Significantly, longitudinal studies also found that children with worse clinical outcomes had reduced FA at baseline than the better outcome group [11], and cross-sectional studies have also found smaller GM and WM volumes in persisters than in remitters [12], which was consistent with smaller brain structures in ADHD_DBD+._ Similarly, although few functional brain studies have been performed in ADHD_DBD+_ patients, Uytun et al. detected higher connectivity in children with ADHD_DBD+_ than in healthy controls [13], which was similarly seen in adults with ADHD [14, 15]. This might suggest that children with ADHD_DBD+_ already exhibit similar brain abnormalities to adult patients as early as childhood, which might be the mechanism behind the persisting ADHD symptoms. However, to date, no studies have simultaneously considered children and adults with ADHD and the effect of DBD symptoms. That is, this conjecture has not been specifically tested.

Over the last decade, the attention of neuroimaging research in ADHD has shifted to the role of distributed neural circuits, and the importance of understanding the function, organization, and development of interacting brain regions has been recognized. Thus, herein, we aim to investigate whether the altered brain functional connectivities (FCs) exhibited in adults with ADHD would be also observed in children with ADHD that co-occurs with disruptive behaviours from quantitative (correlations with disruptive behaviours) and categorical dimensions (comparisons for ADHD with and without DBD). We hypothesized that (1) the more DBD symptoms there are, the more adult-like functional abnormalities there will be in cADHD, and (2) cADHD comorbid DBD might show an adult-like pattern compared with cADHD without DBD. Given the scarcity of studies investigating the differences between ADHD_CD+_ and ADHD_ODD+,_ we did not have a specific prediction for these two groups.

### Methods

Additional file 1: Figure S1 illustrates the whole study flowchart.

#### Subjects and assessment

##### Adults

A total of 138 adults were recruited for the present study. Ninety-eight drug-naïve adults with ADHD (aADHD) (70 males, 28 females; mean age: 27.58 ± 5.42 years; age range: 18–43 years) were recruited from the clinics of Peking University Sixth Hospital/Institute of Mental Health. Forty healthy controls (aHC) (25 males, 15 females; mean age: 27.10 ± 4.63 years; age range: 20–40 years) matched for age, sex, and intelligence quotient (IQ) were recruited from nearby communities and universities.

Conner’s Adult ADHD Diagnostic Interview for Diagnostic and Statistical Manual of Mental Disorders, Fourth Edition (DSM-IV) was used to confirm the diagnosis of ADHD [16] by trained and skilled psychiatrists. The full-scale IQ was assessed using the Wechsler Adult Intelligence Scale-Third Edition. The ADHD Rating Scale-IV (ADHD RS-IV) was completed by all the participants to evaluate the severity of ADHD symptoms (Additional file 1: Table S1).

As in our previous research on adults [17], the inclusion criteria were as follows: (1) aged ≥ 18 years; (2) right-handed; (3) full-scaled IQ ≥ 90; (4) drug-naïve and free of other medical intervention; (5) no history of severe physical disease; and (6) free of a current diagnosis of schizophrenia, severe major depression, clinically significant panic disorder, bipolar disorder, or mental retardation.

##### Children

A total of 172 children with ADHD (cADHD) (161 boys, 11 girls; mean age, 106.48 ± 23.26 months; age range: 72–198 months) were recruited from the clinics of the Peking University Sixth Hospital/Institute of Mental Health. Seventy-seven HC (cHC) (45 boys, 32 girls; mean age, 109.84 ± 10.53 months; age range: 83–135 months) were obtained from a primary school in the local community. The diagnosis and comorbidities of cADHD were diagnosed by an experienced psychiatrist according to the criteria of the DSM-IV by a semistructured interviews using the Clinical Diagnostic Interview Scale (CDIS) [18]. ADHD symptom severity was scored with the ADHD RS-IV [19]. The full-scaled IQ was measured with the Chinese Wechsler Intelligence Scale III for Children (Additional file 1: Table S2).

As in our previous research on children [20], the inclusion criteria were as follows: (1) aged ≤ 16 years; (2) full-scaled IQ ≥ 80; (3) right-handed; (4) psychostimulant-naïve and free of any other medical intervention; and (5) no history of head trauma, neurological illness or other severe diseases such as epilepsy, schizophrenia, pervasive developmental disorders or mental retardation.

The parents of the children with ADHD were asked to fill out the NICHQ Vanderbilt ADHD screen Assessment Scale on a 4-point scale (1 = “never”, 2 = “sometimes,” 3 = “often,” 4 = “always”). The item scores under the oppositional defiant disorder (ODD) and conduct disorder (CD) dimensions were summed separately for further analysis.

#### Functional connectivity analysis

##### Data acquisition

There are various approaches for studying the brain mechanism underlying the pathogenesis of ADHD. Among these, ecologically valid and straightforward measures based on resting-state connectivity with large sample sizes strike a balance between highly specialized paradigms (e.g., task-based) and less-sensitive measures (e.g., structural morphometry) [21]. Functional near-infrared spectroscopy (fNIRS) is a new noninvasive optical brain imaging tool that can be used to measure the hemoglobin concentration changes in the brain related to neural activity. Quantitative studies have demonstrated its reliability and feasibility in characterizing brain activation and functional connectivity [20, 22]. Moreover, with the advantages of high motion tolerance, few body constraints, and portability [23], fNIRS is one of the most suitable tools for studying the brain function of children with ADHD. With FCs and multiscale entropy, our previous works have demonstrated the feasibility and potential of the fNIRS technique in individuals with ADHD [20, 22]. Considering that one recent meta-analysis suggested that ADHD pathophysiology might lie in network interactions rather than regional abnormalities [24], we turned to FCs between brain networks using a resting-state paradigm.

This study used a multichannel continuous-wave near-infrared optical imaging system (Nirscan, Hui Chuang, China) with 24 light sources (wavelengths: 670 and 830 nm) and 28 detectors. It generated 80 measurement channels with a fixed source-detector distance of 3 cm covering the frontal, occipital and parietal lobes. According to the international 10–20 system, the cap was placed with the external auditory canals and vertex as the reference points. Data were collected at a sampling rate of 17 Hz. Each participant underwent an  ~ 12 min brain activity recording while at rest. Participants were asked to sit still and keep their eyes closed without falling asleep. Meanwhile, the surrounding environment remained unchanged.

##### MRI coregistration

To identify the positions of each measurement channel on the brain surface, we randomly selected a child and an adult for structural MRI scanning. The participants lay supine while wearing the fNIRS cap with every channel labeled a vitamin E capsule. Then, their T1-weighted structural image was acquired using a General Electric; Discovery MR750 3.0 Tesla scanner. Next, the MR image was normalized into the Montreal Neurological Institute (MNI) space using SPM12. Then, the MNI coordinates for each channel on the brain scalp were projected to the brain surface via NIRS_SPM to obtain the MNI coordinates of each channel on the brain surface. Finally, the resulting channel coordinates were grouped into different brain networks based on Yeo et al.’s seven network template (Additional file 1: Figure S2) [25].

##### Data preprocessing

We used the FC-NIRS package to preprocess our fNIRS data. First, we removed the channels without a detectable heartbeat component (~ 1 Hz). The raw intensity signals were then converted into optical density signals. Next, we applied the spline interpolation algorithm to the resulting signals to correct the motion artefacts by channels. Motion artefacts were detected over a sliding window of 2 s. Any signal change beyond 5 standard deviations of the entire time series was considered a motion artefact. The resulting signals were then bandpass filtered (0.01–0.1 Hz) to remove the effect of low-frequency drift and high-frequency neurophysiological noise. Next, the relative hemoglobin concentration changes in oxygen-hemoglobin (HbO) and deoxygen-hemoglobin (HbR) were calculated via the modified Beer–Lambert Law. Finally, we extracted a 7-min stable hemoglobin time series for each participant. Of note, the HbO signal was used for the following analysis due to its relatively high signal-to-noise ratio (for more details, please see our previous work [20, 22]).

##### Functional connectivity calculation

The functional connectivity matrix was computed in FC-NIRS, which generated an 80 × 80 correlation matrix for each participant by conducting Pearson correlation analyses between the time series of every pair of channels. We adopted the z matrix (i.e., Fisher’s r-to-z transformation) for the next calculation step due to its normality characteristics. Then, according to MRI coregistration, our measurement channels were grouped into six networks: the visual network (VN), somatomotor network (SMN), dorsal attention network (DAN), ventral attention network (VAN), frontoparietal network (FPN), and default mode network (DMN). After considering the brain hemisphere factor, we obtained 12 networks in total. Finally, to evaluate the FCs between and within networks, the z values of the functional connectivity matrix were averaged separately, resulting in a 12*12 functional connectivity matrix.

#### Statistical analysis

##### FC differences in adults

Based on our conjecture, we first calculated the difference between aADHD and aHC. Differences in age, IQ, sex, and core symptoms between aADHD and aHC were estimated using two-sample t tests or chi-square tests (i.e., sex variables). The differences in FCs were determined using a univariate general linear model (GLM), with age, IQ, and sex as covariates. The FDR correction method was used for multiple comparisons (*p*_FDR_ < 0.05).

##### FC differences in children

Similarly, differences in age, IQ, sex, and core symptoms between cADHD and cHC were estimated using two-sample t tests or chi-square tests (i.e., sex variable). Then, for the abnormal FCs in aADHD, we compared their differences between cADHD and cHC using a univariate GLM, with age, IQ, and sex as covariates. The FDR correction method was used for multiple comparisons (*p*_FDR_ < 0.05).

##### The relationship between altered FCs and disruptive behaviours in cADHD

The FCs exhibiting significant group differences in aADHD were first marked, and the corresponding FCs in children were extracted from their individual FCs matrix. Then, the covariate-adjusted Spearman’s rank correlation was estimated between these FCs and disruptive symptoms assessed by CD/ODD scores from the NICHQ Vanderbilt ADHD screen Assessment Scale.

##### FC differences in ADHD_CD+_/ADHD_ODD+_, ADHD_CD__−_/ADHD_ODD__−_ and cHC

Finally, to render the results readily interpretable, we compared the FC differences among ADHD_CD−_, ADHD_CD+_ and cHC groups and among ADHD_ODD−_, ADHD_ODD+_ and cHC groups. Considering the relatively low incidence rate of ADHD_CD+_, we defined subthreshold ADHD_CD+_ for group comparison analyses. The detailed information is described in Additional file 1: Appendix S2. Considering the mismatch of sample size between ADHD_CD+_/ADHD_ODD+_ (mainly for the subthreshold ADHD_CD+_) and cHC, we randomly selected one group of cHC that was 1:1 sex and age- matched with ADHD_CD+_/ADHD_ODD+_ using R.

The group differences in IQ and ADHD core symptoms were compared using one-way ANOVA. FC differences were detected using univariate GLM, with age, sex, and IQ as covariates.

##### Sensitivity analysis

To rule out the potential influence of other comorbidities and core symptoms on our results, we included comorbidities other than CD/ODD and total symptoms as covariates and repeated the analyses.

### Results

#### Demographic and clinical variables of adults

Group comparisons in the demographic and clinical variables between aADHD and aHC are listed in Additional file 1: Table S1. There were no significant differences in age, sex, or IQ, whereas the aADHD group exhibited higher scores of core symptoms.

#### Statistical difference in FCs between aADHD and aHC

**Fig. 1:**
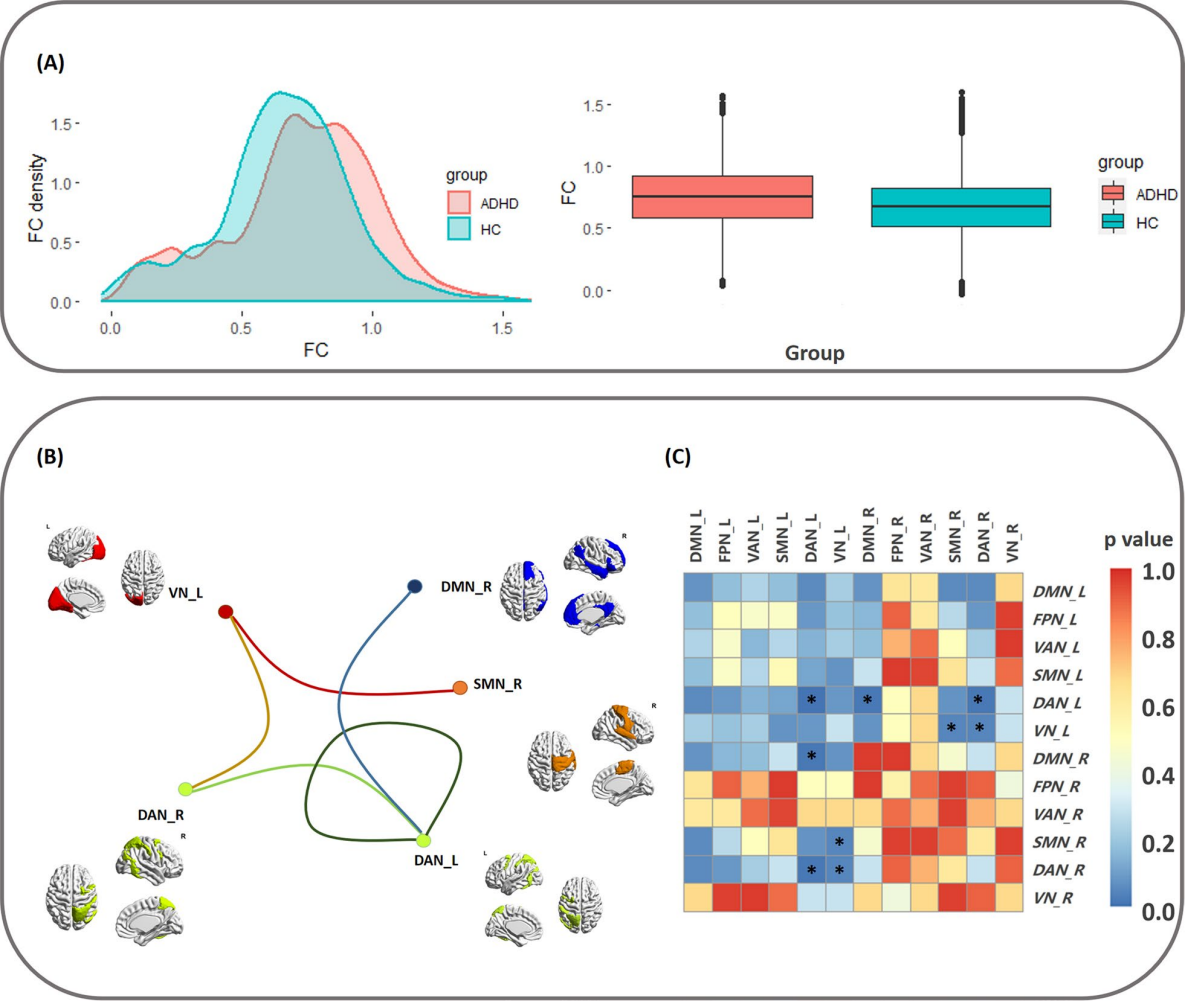
Distribution and group difference of FC in different brain networks of adults. **A** Histograms and boxplot of the functional connectivity distribution in adults. aADHD have larger FCs (0.73 ± 0.28) values than aHC (0.65 ± 0.25). **B** The difference in FC between aADHD and aHC. Lines indicate statistically significant increase in value (p < 0.05, after FDR correction). **C** Heatmap shows the difference in FC between aADHD and aHC. Black stars indicate a statistically significant decrease in value (p < 0.05). *VN,* visual network; *SMN*, somatomotor network; *DAN,* dorsal attention network; *VAN,* ventral attention network; *FPN,* frontoparietal network; *DMN,* default mode network; *L,* left; *R,* right

We first averaged the functional connectivity matrix to obtain the mean connectivity strength for each participant and then compared the group differences. We found that the aADHD group (0.73 ± 0.28) exhibited increased FCs compared with the aHC group (0.65 ± 0.25), albeit not significantly (*p* = 0.142) (Fig. 1A). A precise examination of FC revealed that the aADHD group exhibited increased FCs in the following: DAN(L)-DAN(L), VN(L)-SMN(R), VN(L)-DAN(R), DAN(L)-DAN(R) and DAN(L)-DMN(R) (Fig. 1B, 1C).

#### Demographic and clinical variables of children

Group comparisons of the demographic and clinical variables between cADHD and cHC are listed in Additional file 1: Table S2. There were no significant differences in age, whereas the cHC group had a significantly higher IQ and more girls. Similarly, the cADHD group exhibited higher scores of ADHD core symptoms.

#### Statistical difference in FCs between cADHD and cHC

The abnormal FCs indicated in aADHD were all reduced in the cADHD group compared with cHC after adjusting for age, sex, and IQ (Additional file 1: Table S3), which were opposite to those in aADHD.

#### The relationship between altered FCs and disruptive behaviours in cADHD

**Fig. 2:**
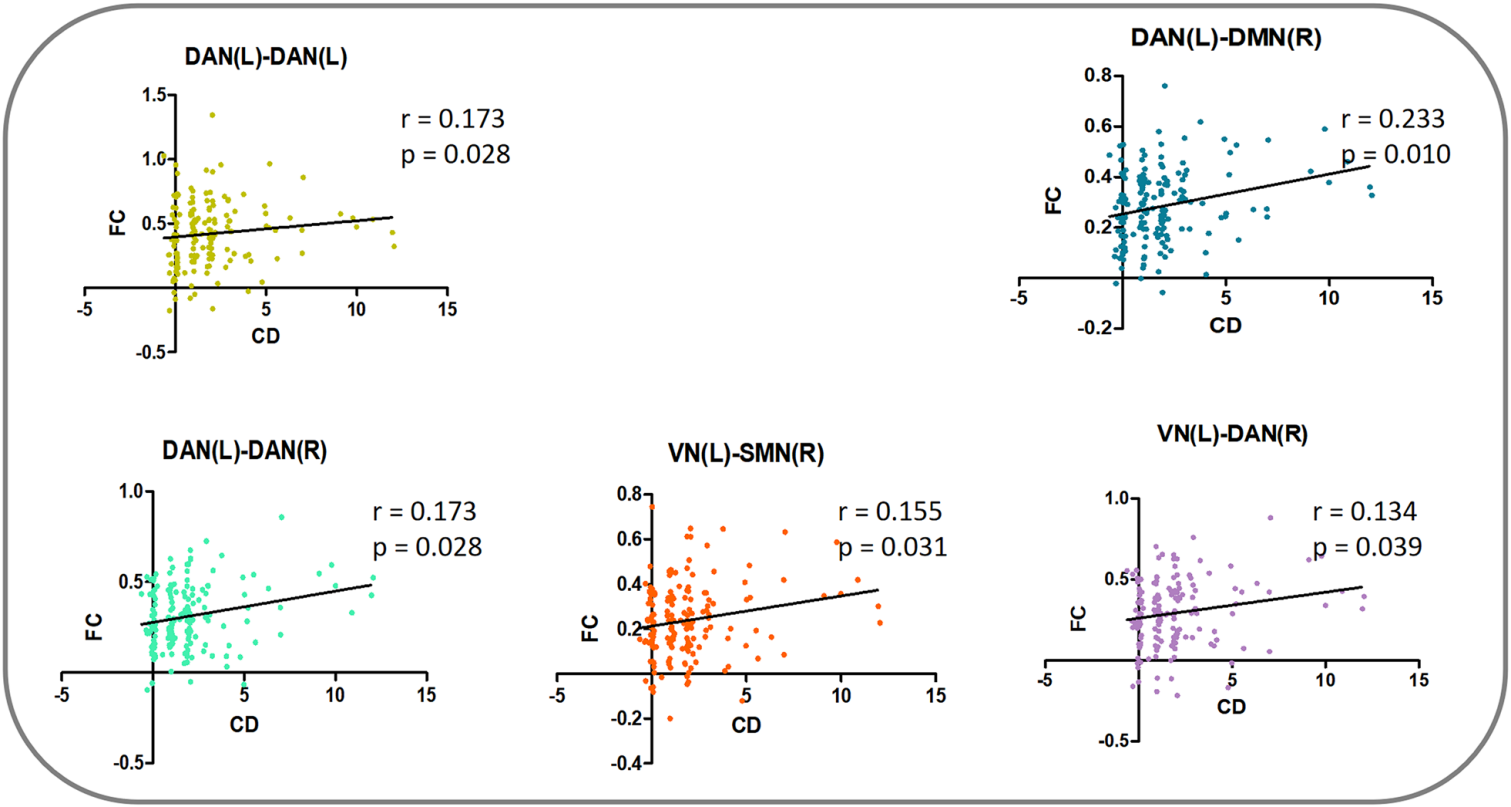
The relationship between altered FCs and disruptive behaviours in cADHD. The scatter plots show a correlation between the CD scores of children with ADHD and the FC in different brain networks. Partial r and *p* values were obtained after adjustment for age, sex and IQ. *DMN,* default mode network; *FPN*, frontoparietal network; *VAN,* ventral attention network; *SMN,* somatomotor network; *DAN,* dorsal attention network; *VN*, visual network; *L,* left; *R,* right

For the FCs showing group differences in adults, their correlation with CD and ODD symptoms in cADHD was explored. We found a significant positive correlation between CD and all these FCs. However, no significant correlation was found between FCs and ODD total symptoms (Table 1, Fig. 2).

**Table 1:** Correlation between FCs and CD, ODD symptoms in cADHD

FC	CD		ODD	
	*r*	*p*_FDR_	*r*	*p*_FDR_
DAN(L)-DAN(L)	0.173	0.028	− 0.017	0.418
DAN(L)-DMN(R)	0.233	0.010	0.053	0.335
DAN(L)-DAN(R)	0.173	0.028	0.096	0.335
VN(L)-SMN(R)	0.155	0.031	0.082	0.335
VN(L)-DAN(R)	0.134	0.039	0.049	0.335

Covariate-adjusted Spearman’s Rank Correlation, one-tailed, after adjusting for age, gender, and IQ; FDR correction

*VN* visual network; *SMN* somatomotor network; *DAN* dorsal attention network; *VAN* ventral attention network; *DMN* default mode network; *L* left; *R* right

In addition, to further supplement the results, we also provided the differences in FCs of the whole brain, and explored their relationship with CD/ODD symptoms. The results indicated widespread reduction in network connectivity in cADHD compared with cHC (See Additional file 1: Figure S3). Further analyses showed significantly positive correlation between CD symptoms and FCs in multiple brain networks, including the five aADHD-altered FCs (Additional file 1: Figure S4A). For ODD symptoms, some positive correlation was indicated in some networks, however none of them overlapped with the abnormal network connections in adults (Additional file 1: Figure S4B). Besides, the correlation coefficients between ODD symptoms and FCs were lower than that between CD symptoms and FCs.

#### Categorial analyses to compare the FCs of ADHD_CD+_/ADHD_ODD+_, ADHD_CD__-_/ADHD_ODD__-_ and cHC

##### ADHD_CD−_, subthreshold ADHD_CD+_ and cHC

**Fig. 3:**
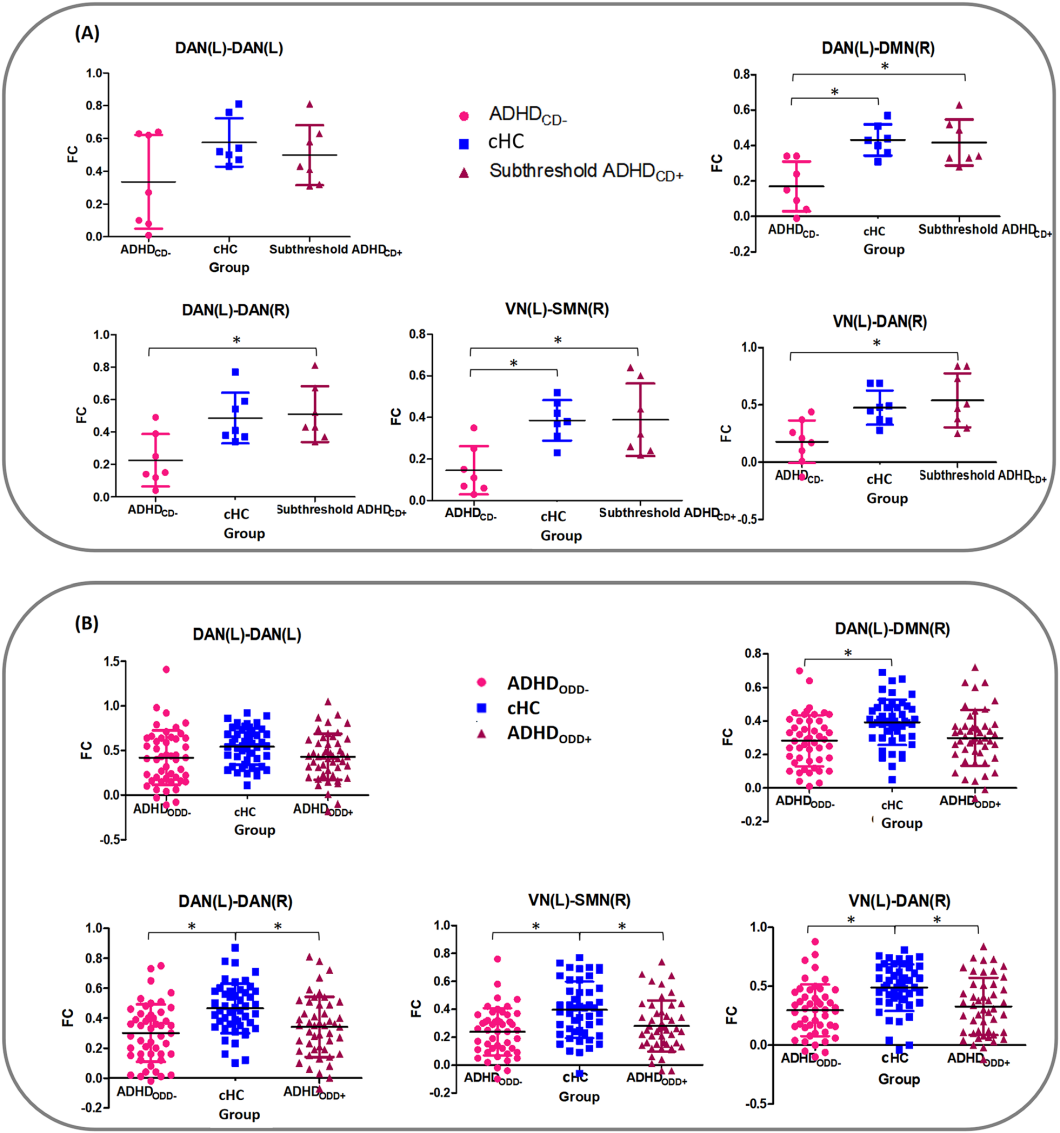
FC difference of ADHD_CD−_/ADHD_ODD−_, ADHD_CD−_/ADHD_ODD−_, and cHC. **A** FC of ADHD_CD−_, subthreshold ADHD_CD+_ and cHC from ANOVA for group differences with individual mean values and standard deviation (SD). **B** FC of ADHD_ODD−,_ ADHD_ODD+_ and cHC from ANOVA for group differences with individual mean values and standard deviation (SD). *DMN,* default mode network; *FPN,* frontoparietal network; *VAN,* ventral attention network; *SMN,* somatomotor network; *DAN,* dorsal attention network; *VN,* visual network; *L,* left; *R,* right. *p < 0.05

Detailed information on the demographic variables is presented in Additional file 1: Table S4. For FC differences, we found significant group differences in DAN(L)-DMN(R), DAN(L)-DAN(R), VN(L)-SMN(R), and VN(L)-DAN(R). For FCs of DAN(L)-DMN(R) and VN(L)-SMN(R), post hoc comparisons showed decreased FCs in ADHD_CD−_ compared with cHC and subthreshold ADHD_CD+_. For FCs of AN(L)-DAN(R) and VN(L)-DAN(R), post hoc comparisons showed decreased FCs in ADHD_CD−_ compared with subthreshold ADHD_CD+_. For the comparison between subthreshold ADHD_CD+_ and cHC, the mean values of FCs in subthreshold ADHD_CD+_ were visually larger (the number is larger) than those of cHC in DAN(L)-DAN(R), VN(L)-SMN(R), and VN(L)-DAN(R), although these difference was not statistically significant. Descriptive statistics can be found in Additional file 1: Table S5 and Fig. 3A.

##### ADHD_ODD__−_, ADHD_ODD+_ and cHC

The demographics and clinical characteristics of the samples are summarized in Additional file 1: Table S6. For group comparisons of FCs, we found that there were significant group differences in the FCs of DAN(L)-DMN(R), DAN(L)-DAN(R), VN(L)-SMN(R), and VN(L)-DAN(R). Post hoc comparisons showed decreased FCs in ADHD_ODD−_ and ADHD_ODD+_ compared with cHC, but there was no difference between ADHD_ODD+_ and ADHD_ODD−_ in the DAN(L)-DAN(R), VN(L)-SMN(R) and VN(L)-DAN(R). In DAN(L)-DMN(R), post hoc comparisons showed decreased FCs in ADHD_ODD−_ compared with cHC (Additional file 1: Table S7, Fig. 3B).

##### Sensitivity analysis

The results did not change significantly after controlling for other comorbidities and core symptoms (Additional file 1: Tables S8–S12).

## Discussion

This study aimed to investigate whether functional brain alterations in aADHD could also be found in cADHD co-occurring with DBD behaviours from quantitative and categorical dimensions. Consistent with previous studies, our results found that aADHD have aberrant brain function in multiple brain areas compared with aHC. More importantly, in cADHD, the aforementioned abnormal FCs in these areas were also detected, however, in an opposite orientation. In further analyses for disruptive behaviours, these altered FCs all indicated a positive association with CD symptoms in cADHD. In addition, the subthreshold ADHD_CD+_ group even exhibited a tendency of adult-like increased FCs in some of the brain networks.

ADHD is a neurodevelopmental disorder diagnosed in children before 12 years. Approximately 15% of youths with ADHD still meet full diagnostic criteria in adulthood, leading to functional impairment in their daily lives. Our study showed that aADHD exhibited increased FCs compared with aHC. Interestingly, the CD score has a positive correlation with FCs of these areas in cADHD. In addition, in the comparison of subthreshold ADHD_CD+_, ADHD_CD−_ and cHC, we found that ADHD_CD−_ had significantly decreased FCs compared with subthreshold ADHD_CD+_ and cHC, while no difference was found between subthreshold ADHD_CD+_ and cHC. Rather, there was even a tendency for an increase in subthreshold ADHD_CD+_ compared with cHC, which was quite similar to the functional abnormalities in adults. Notably, due to the low incidence of CD in children with ADHD, we chose “subthreshold ADHD_CD+_”, which means that these children cannot fully meet the diagnosis of CD in NICHQ Vanderbilt. Nevertheless, we found that the mean value of subthreshold ADHD_CD+_ was larger than that of cHC. Therefore, we speculate that if we include a large sample size of cADHD patients who satisfied the criteria of CD in NICHQ Vanderbilt, ADHD_CD+_ might show adult-like significantly increased FCs in these areas compared with cHC. In addition, to further supplement the results in the present study, we also analysed the difference in FCs between cADHD and cHC of all brain networks. We found that cADHD showed widespread reductions in network connectivity compared with cHC. Taken together with the observed increased FCs in aADHD, this might suggest that the developmental delay in cADHD might gradually improve during the transition from childhood to adulthood, and some specific FCs might even show compensatory enhancement. Among these cADHD-reduced FCs, some showed a strong correlation with CD symptoms, including the five aADHD-altered FCs. Notably, these aberrant FCs in the current study were located in the VN, DMN, SMN, and DAN, which are associated with visual sensory processing [26], higher-order cognition [27], sensorimotor functions [28], and top-down attentional control [29] that have long been considered to be involved in ADHD [30]. In summary, these findings suggest that cADHD with CD already display adult-like abnormalities in the key injured brain networks of aADHD as early as childhood.

Recently, one large-scale study of 17,075 individuals found that age and cortical thickness showed a negative association [31]. While previous studies have shown that ADHD_DBD+_ have significantly smaller cortical thickness compared with ADHD_only_ [32]. Thus, it might also suggest that ADHD_DBD+_ might be a more “mature state” than ADHD_only_. In addition, in neuropsychological functioning, a previous study found that ADHD_ODD+_ individuals showed more deficits than cHC in verbal memory and response inhibition, but ADHD_CD+_ individuals did not differ from cHC in neuropsychological function [33]. ADHD_CD+_ showed no significant difference from cHC, while ADHD_ODD+_ and ADHD_only_ exhibited decreased FCs compared with cHC, which agrees with the neuropsychological study mentioned. Thus, we suspect that the increased FCs in ADHD_CD+_ might be due to the compensatory response. To reduce the impairment from CD, their brain function turns to a “mature” state (relative to the developmental delay of cADHD [34]), and this pattern of compensation might “carry forward” and eventually lead to persistence of ADHD symptoms.

In contrast, there was no significant correlation between ODD symptoms and these FCs. The ADHD_ODD+_ subgroup showed decreased FCs compared with cHC, which was different from the increased FCs in the subthreshold ADHD_CD+_ group. Interestingly, Caye et al. carried out the first meta-analysis of longitudinal studies assessing the risk markers for the persistence of ADHD and found that comorbid CD emerged as a predictor for ADHD persistence from childhood to adulthood. However, comorbid ODD was investigated by four studies with divergent results [35]. Similarly, one 10-year follow-up study showed that only ADHD_CD+_ was associated with multiple adverse outcomes, including bipolar, psychoactive substance use disorders, and smoking [36]. Furthermore, unlike ODD, CD includes aggression towards people and animals or property destruction. In addition, previous studies proposed that CD from childhood onwards is a more harmful condition and is considered less receptive to intervention than ODD [37]. Children with ADHD_ODD+_ may form an intermediate subgroup between ADHD_only_ and ADHD_CD+_ [38, 39]. Some scholars even suggested that ODD is a common feature that is exaggerated in normal adolescents, and it should be considered a temperament dimension rather than a separate categorical disorder [3]. Therefore, it is not surprising that although the association between ODD and FCs indicated the same association trend as CD, this correlation was not significant.

At the neuroimaging level, most studies included mixed samples of cADHD comorbid with both ODD and CD. Although only a few small studies distinguished ADHD_CD+_ from ADHD_ODD+_, most of them still revealed a significant difference between ADHD_CD+_ and ADHD_ODD+_. For example, van Ewijk et al. found that comorbid ODD is associated with altered WM microstructure, and there was an interaction between ODD and (subclinical) CD, which indicated that ODD and CD should be treated as separate constructs [40]. This again illustrates that comorbid ODD and comorbid CD are different in children with ADHD. At present, there is only one resting-state fMRI study of ADHD_CD+_. Interestingly, although most task fMRI studies found decreased DMN activity in CD-only adolescents [41, 42], this study shows that DMN-related FCs were increased in ADHD_CD+_ compared with cHC [13]. The authors proposed that this phenomenon could be similar to the hypothesis that ADHD may have increased intrusions during task performance that are displayed as lapses of attention and variable patterns of response, which partly reflect improper deactivation of the DMN [43]. These studies all highlight the importance of distinguishing between ADHD_CD+_ and ADHD_ODD+_ from a etiological insight.

## Limitations

Our current findings should be viewed in light of some limitations. First, fNIRS can only examine the cortical surface within 2–3 cm of the cortex. Deep structures (e.g., the hippocampus or amygdala) cannot be measured with fNIRS [44]. ADHD neuroimaging studies have shown deficits in subcortical regions, such as the basal ganglia and insula. Neuroimaging studies in children with CD also revealed a smaller size of the subcortex, including the amygdala and insula [45]. Thus, studies on the subcortex may shed more light on our findings in the future. Second, the number of ADHD_CD+_ patients was relatively small. Although we used “subthreshold ADHD_CD+_,” we only obtained 7 children who met the criteria. However, considering the robust findings from quantitative analyses for CD symptoms, we anticipate that further categorical studies with a larger sample size will exhibit more compelling results. Similarly, the number of samples from female subjects is low in children due to the lower prevalence of ADHD in females. Future studies with large sample sizes are needed to validate our results. Third, this work is a cross-sectional study. It is unclear whether ADHD_CD+_ in children would certainly persist throughout development and into adulthood. In addition, we should note that ADHD_CD+_/ADHD_ODD+_ in children may continue to develop into ODD and/or CD in the future. Thus, longitudinal studies are required to verify the mechanism in the future.

## Conclusion

Using resting-state fNIRS data, we investigated the relationship between aADHD-related functional abnormalities and disruptive behaviours in cADHD. This study suggested that CD symptoms in children with ADHD, rather than ODD, could be more closely correlated with the risk of persisting ADHD from a neurobiological perspective. Our work provides some evidence for the brain development of ADHD. This highlighted the importance of differentiating ADHD_CD+_ from ADHD, and children with this condition should receive more specialized care, as they are more likely to persist with throughout development.

## Supplementary Information

**Additional file 1: Appendix S1.** Tables and Figures. **Appendix S2.** FCs differences of ADHD_CD+_/ADHD_ODD+_, ADHD_CD__−_/ADHD_ODD-_ and cHC.

## Data Availability

Due to the nature of this research, participants of this study did not agree for their data to be shared publicly, so supporting data is not available.
