# Supplementary material for: The potential shared brain functional alterations between adults with ADHD and children with ADHD co-occurred with disruptive behaviors
Source: Child Adolesc Psychiatry Ment Health. 2022 Jun 27;16:54. doi: 10.1186/s13034-022-00486-7 (PMC9238266; doi:10.1186/s13034-022-00486-7)
Supplement: Supplementary file 1 — Additional file 1: Appendix S1. Tables and Figures. Appendix S2. FCs differences of ADHDCD+/ADHDODD+, ADHDCD−/ADHDODD- and cHC. [file 13034_2022_486_MOESM1_ESM.docx]

**Supplementary**

# Appendix 1. Tables and Figures


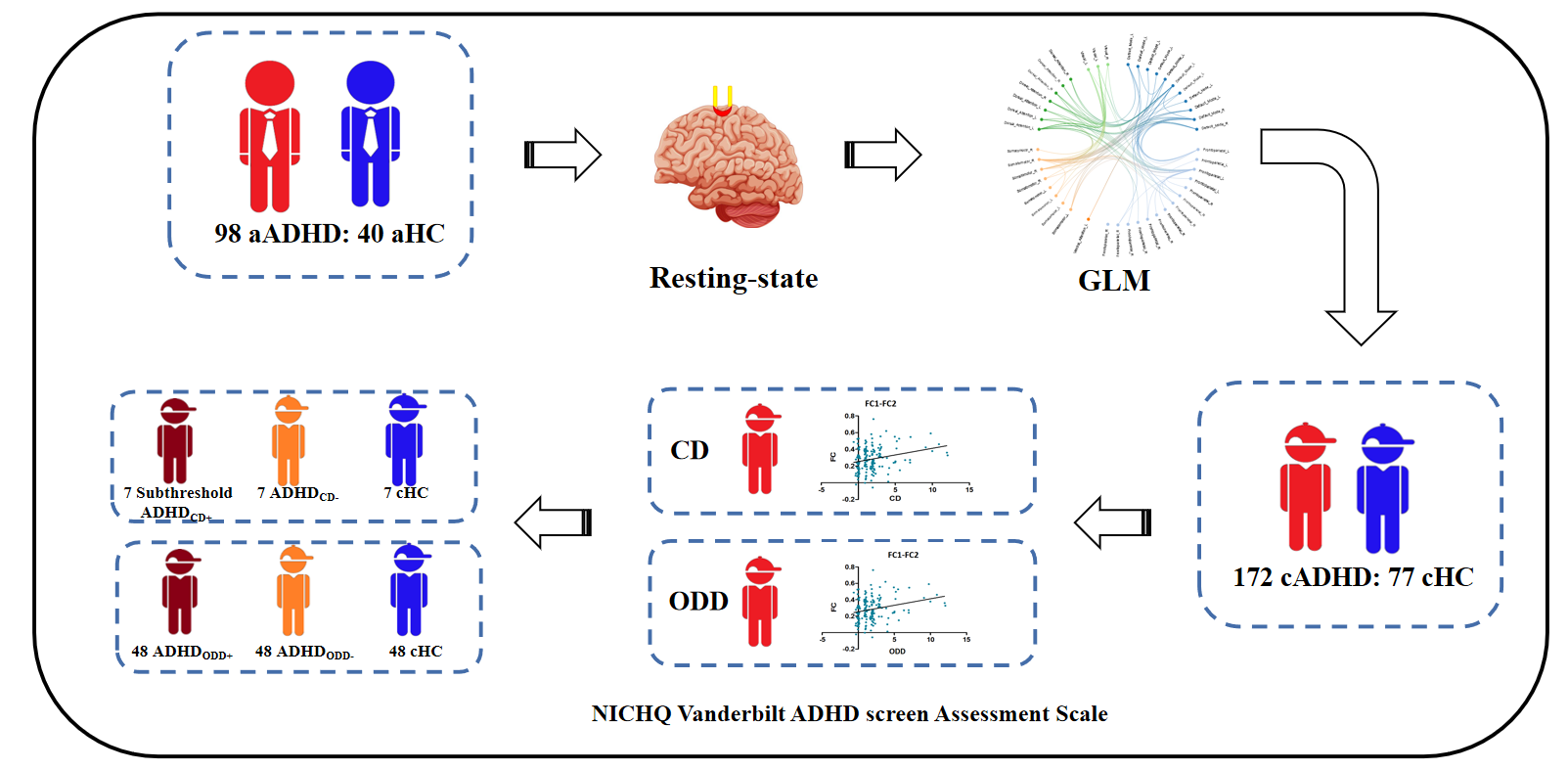


**Fig S1. Flowchart of the whole study design.**


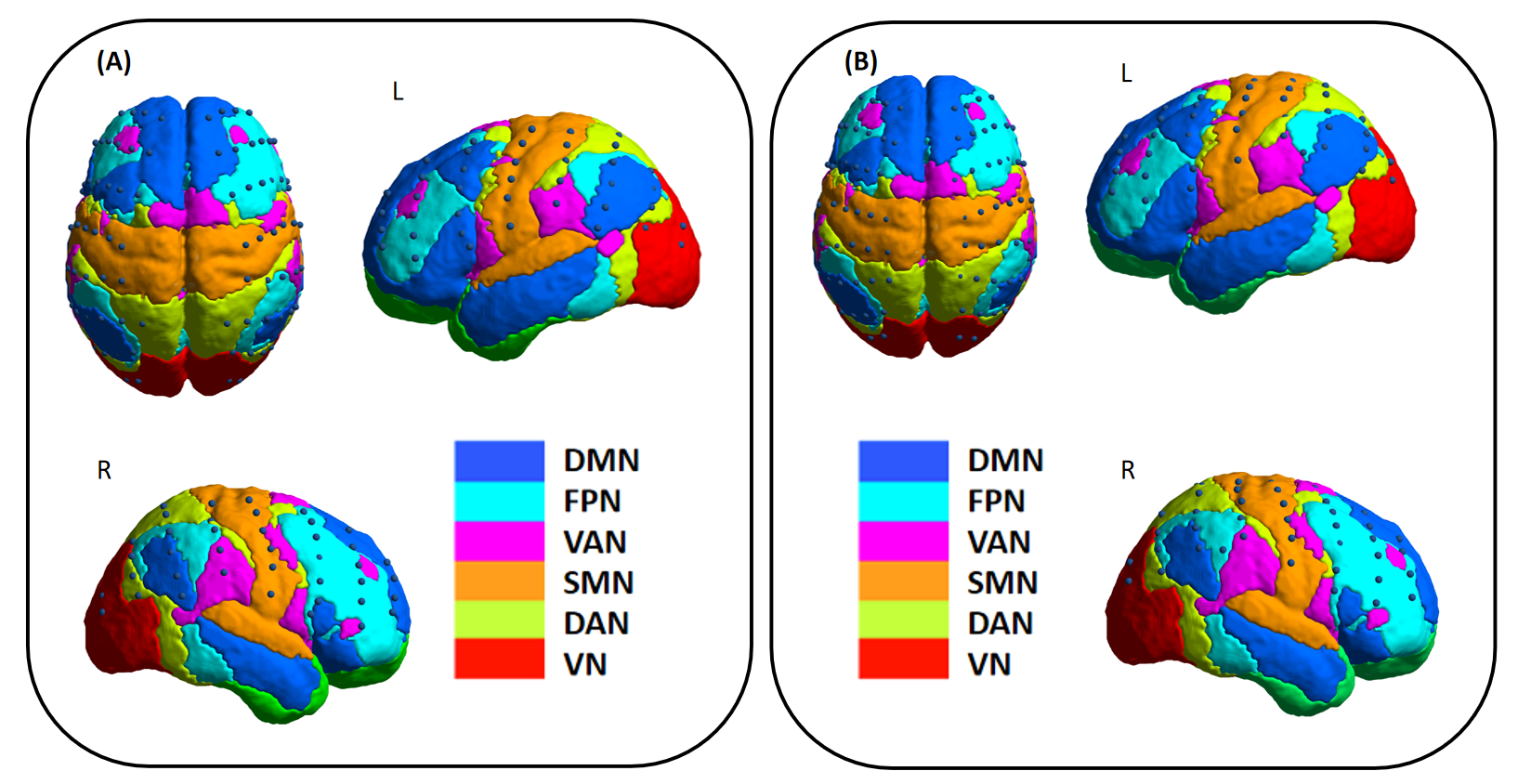


**Fig S2. Arrangement of channels in children and adults.**

(A) The arrangement of the whole-brain 80 measurement channels on Yeo’s brain template in children; (B) The arrangement of the whole-brain 80 measurement channels on Yeo’s brain template in adults.


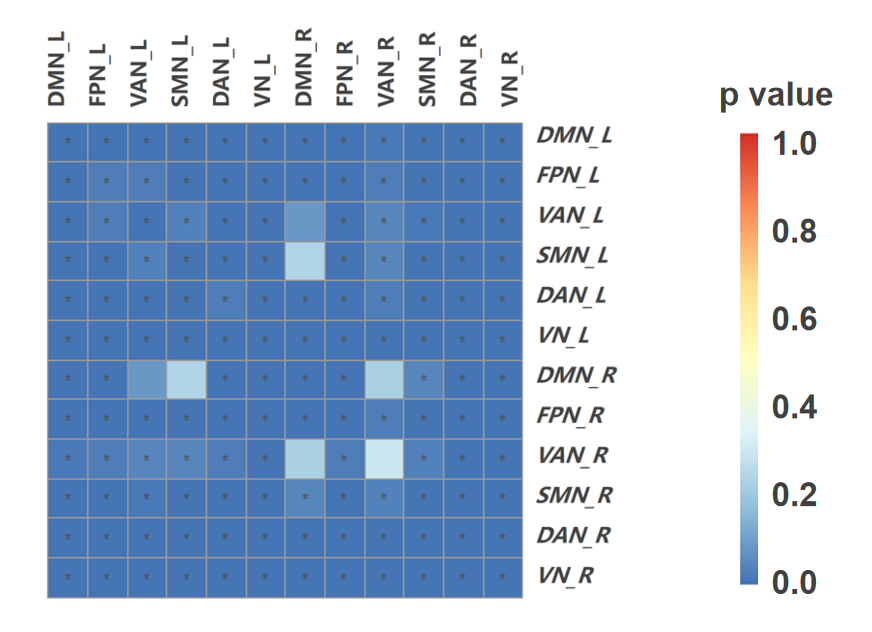


**Figure S3. The difference in FCs between ADHD and HC in children.**

* Statistically significant decreased in value (p < 0.05, after FDR correction).


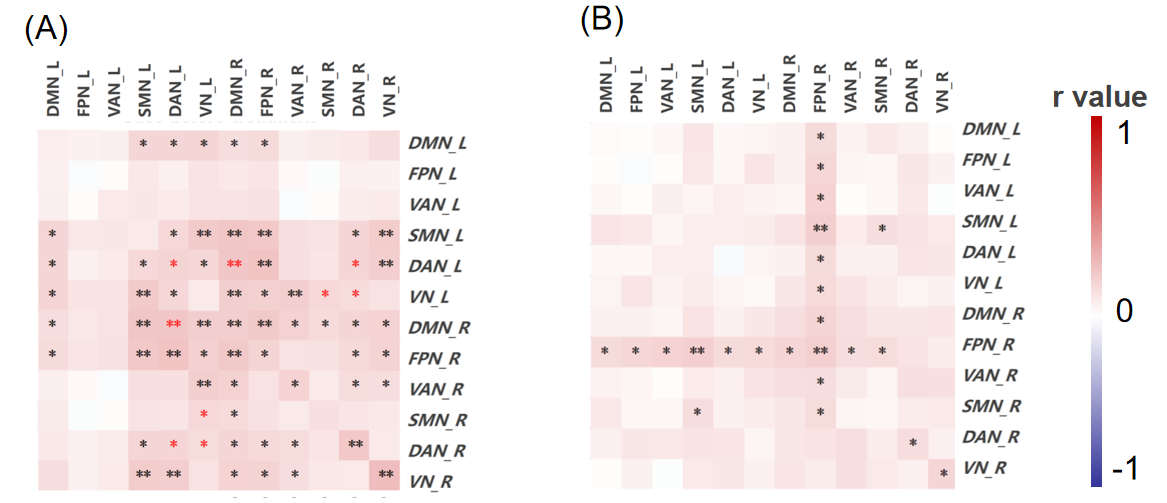


**Figure S4. The correlation between CD/ODD symptoms and FCs in children with ADHD.**

(A)The correlation between CD and FCs in children with ADHD；(B)The correlation between ODD and FCs in children with ADHD. Red asterisks indicates the FCs which were altered in adults with ADHD. ** p<0.001; * p<0.05 (before FDR correction).

**Table S1. Sample characteristics of adults**

| **Characteristic** | **aADHD (n = 98)** | **aHC (n = 40)** | ***x*^2^/*t*** | ***p*** |
| --- | --- | --- | --- | --- |
| **Sex (M:F)** | 70:28 | 25:15 | 1.06 | 0.304 |
| **Age (mean ± SD)** | 27.58 ± 5.42 | 27.10 ± 4.63 | 0.49 | 0.623 |
| **IQ (mean ± SD)** | 122.19 ± 8.16 | 121.88 ± 7.97 | 0.21 | 0.833 |
| **ADHD subtype** |  |  |  |  |
| **C/IA** | 26/72 | - | - | - |
| **ADHD symptoms (mean ± SD)** |  |  |  |  |
| **Inattentive** | 26.16 ± 6.80 | 12.68 ± 4.54 | 11.52 | <0.001 |
| **Hyperactive/impulsive** | 19.98 ± 6.19 | 11.89 ± 2.19 | 11.06 | <0.001 |
| **Total** | 47.26 ± 8.84 | 25.24 ± 4.97 | 18.09 | <0.001 |

**Abbreviation:** ADHD = Attention-deficit/hyperactivity disorder; IQ = intelligence quotient; C/IA= Combined subtype/Inattentive subtype;

**Table S2. Sample characteristics of children**

| **Characteristic** | **cADHD (n = 172)** | **cHC (n = 77)** | ***x*^2^/*t*** | ***p*** |
| --- | --- | --- | --- | --- |
| **Sex (M:F)** | 161:11 | 45:32 | 46.03 | <0.001 |
| **Age (mean ± SD)** | 106.48 ± 23.26 | 109.84 ± 10.53 | -1.57 | 0.118 |
| **IQ (mean ± SD)** | 107.92 ± 13.88 | 115.40 ± 11.22 | -4.15 | <0.001 |
| **ADHD subtype** |  |  |  |  |
| **C/IA/HI/NOS** | 72/88/6/6 | - | - | - |
| **ADHD symptoms (mean ± SD)** |  |  |  |  |
| **Inattentive** | 26.33 ± 4.88 | 8.26 ± 4.75 | 26.86 | <0.001 |
| **Hyperactive/impulsive** | 21.77 ± 6.17 | 6.82 ± 4.15 | 22.02 | <0.001 |
| **Total** | 48.10 ± 8.99 | 14.69 ± 8.16 | 28.67 | <0.001 |

**Abbreviation:** ADHD = Attention-deficit/hyperactivity disorder; IQ = intelligence quotient; C/IA /HI/NOS= Combined subtype/Inattentive subtype/Hyperactive and impulsive subtype/ADHD-not otherwise specified

**Table S3. Comparison of FC in cADHD and cHC**

| **Dependent variables** | **cADHD**  **(mean ± SD)** | **cHC**  **(mean ± SD)** | **F** | ***P_FDR_**** |
| --- | --- | --- | --- | --- |
| **DAN(L)-DAN(L)** | 0.42 ± 0.265 | 0.57 ± 0.246 | 6.07 | 0.014 |
| **DAN(L)-DMN(R)** | 0.28 ± 0.153 | 0.39 ± 0.141 | 12.10 | 0.001 |
| **DAN(L)-DAN(R)** | 0.31 ± 0.190 | 0.47 ± 0.166 | 20.46 | <0.001 |
| **VN(L)-SMN(R)** | 0.24 ± 0.176 | 0.38 ± 0.185 | 21.71 | <0.001 |
| **VN(L)-DAN(R)** | 0.29 ± 0.220 | 0.48 ± 0.193 | 25.55 | <0.001 |

**Note:** *After adjusting for age, sex, and IQ; *P* values after FDR corrected.

**Abbreviation:** ADHD = Attention deficit hyperactivity disorder; HC = healthy control; VN = visual network; SMN = somatomotor network; DAN = dorsal attention network; VAN = ventral attention network; DMN = default mode network; L = left; R = right.

**Table S4. Sample characteristics of ADHD_CD-_, ADHD_CD+_ and cHC**

| **Characteristic** | **ADHD_CD -_(n = 7)** | **cHC (n = 7)** | **Subthreshold ADHD_CD+_**  **(n = 7)** | ***F*** | ***p*** | **Post hoc comparisons**  **(p value)** |
| --- | --- | --- | --- | --- | --- | --- |
| **Sex (M:F)** | 7:0 | 7:0 | 7:0 | - | - | - |
| **Age (month; mean ± SD)** | 109.57 ± 10.50 | 114.71 ± 5.31 | 102.29 ± 17.41 | 1.85 | 0.185 | - |
| **IQ (mean ± SD)** | 102.51 ± 8.26 | 119.89 ± 2.70 | 103.29 ± 11.90 | 9.28 | 0.002 | a(0.004)  c(0.005) |
| **Subtype** |  |  |  |  |  |  |
| **IA/H/C** | 4/0/3 | / | 1/0/6 | - | - | - |
| **ADHD symptoms (mean ± SD)** |  |  |  |  |  |  |
| **Inattentive** | 26.43 ± 4.96 | 9.29 ± 4.54 | 20.14 ± 4.56 | 39.39 | <0.001 | a(<0.001)  c(<0.001) |
| **Hyperactive/impulsive** | 23.43 ± 4.08 | 9.71 ± 4.89 | 29.57 ± 4.78 | 36.91 | <0.001 | a(<0.001)  c(<0.001) |
| **Total** | 49.86 ± 8.09 | 19.00 ± 9.24 | 59.71 ± 7.83 | 44.68 | <0.001 | a(<0.001)  c(<0.001) |

**Note:** Post hoc comparisons were corrected with Bonferroni–Holm method; a, ADHD_CD-_ versus HC; b, ADHD_CD-_ versus ADHD_CD+_; c , HC versus ADHD_CD+._

**Abbreviation:** ADHD = Attention deficit hyperactivity disorder; ADHD_CD_- = ADHD children, none of the CD item of NICHQ Vanderbilt ADHD screen were greater or equal to 2; ADHD_CD+_ = ADHD children, at least 2 CD items of NICHQ Vanderbilt ADHD screen were greater or equal to 2; HC = healthy control; IQ = Estimated intelligence quotient; IA/H/C= Inattentive/Hyperactive-impulsive/Combinedsubtype.

**Table S5. Comparison of FC in ADHD_CD-_, ADHD_CD+_ and cHC**

| **Dependent variables** | **ADHD_CD-_**  **(mean ± SD)** | **cHC**  **(mean ± SD)** | **Subthreshold ADHD_CD+_**  **(mean ± SD)** | ***F*** | **Adjusted *p*** | **Post hoc comparisons**  **(*p* value)** |
| --- | --- | --- | --- | --- | --- | --- |
| **DAN(L)-DAN(L)** | 0.3357 ± 0.2862 | 0.5757 ± 0.1480 | 0.4986 ± 0.1830 | 1.430 | 0.268 | / |
| **DAN(L)-DMN(R)** | 0.1671 ± 0.1440 | 0.4314 ± 0.0878 | 0.4171 ± 0.1298 | 11.714 | 0.001 | a(0.023)  b(0.001) |
| **DAN(L)-DAN(R)** | 0.2171 ± 0.1740 | 0.4857 ± 0.1561 | 0.5100 ± 0.1718 | 6.872 | 0.007 | b(0.006) |
| **VN(L)-SMN(R)** | 0.1429 ± 0.1193 | 0.3857 ± 0.097 | 0.3886 ± 0.1743 | 10.743 | 0.001 | a(0.025)  b(0.001) |
| **VN(L)-DAN(R)** | 0.1800 ± 0.2010 | 0.4914 ± 0.1542 | 0.4971 ± 0.2185 | 6.211 | 0.010 | b(0.013) |

**Note:** After adjusting for age, sex, and IQ; Post hoc comparisons were corrected with Bonferroni–Holm method; a, ADHD_CD-_ versus HC; b, ADHD_CD-_ versus ADHD_CD+_; c, HC versus ADHD_CD+._

**Abbreviation:** ADHD = Attention deficit hyperactivity disorder; ADHD_CD_- = ADHD children, none of the CD item of NICHQ Vanderbilt ADHD screen were greater or equal to 2; ADHD_CD+_ = ADHD children, at least 2 CD items of NICHQ Vanderbilt ADHD screen were greater or equal to 2; HC = healthy control; VN = visual network; SMN = somatomotor network; DAN = dorsal attention network; VAN = ventral attention network; DMN = default mode network; L = left; R = right.

**Table S6. Sample characteristics of** **ADHD_ODD-_, ADHD_ODD+_ and cHC**

| **Characteristic** | **ADHD_ODD-_**  **(n = 48)** | **cHC**  **(n = 48)** | **ADHD_ODD+_**  **(n = 48)** | **F value** | ***p*** | **Post hoc comparisons**  **(*p* value)** |
| --- | --- | --- | --- | --- | --- | --- |
| **Sex (M:F)** | 46:2 | 46:2 | 46:2 | - | - | - |
| **Age (month; mean ± SD)** | 109.15 ± 24.57 | 113.04 ± 11.02 | 110.63 ± 26.04 | 0.40 | 0.673 | - |
| **IQ (mean±SD)** | 106.08 ± 14.19 | 113.95 ± 12.29 | 106.50 ± 13.71 | 5.22 | 0.007 | a(0.014) |
| **Subtype** |  |  |  |  |  |  |
| **IA/H/C** | 19/1/28 | / | 34/4/10 | - | - | - |
| **ADHD symptoms (mean ± SD)** |  |  |  |  |  |  |
| **Inattentive** | 25.77 ± 3.50 | 9.24 ± 5.19 | 27.90 ± 3.95 | 268.14 | <0.001 | a(<0.001)  b(0.047)  c(<0.001) |
| **Hyperactive/impulsive** | 19.73 ± 5.16 | 7.13 ± 4.58 | 24.48 ± 6.29 | 128.74 | <0.001 | a(<0.001)  b(<0.001)  c(<0.001) |
| **Total** | 45.50 ± 5.81 | 16.37 ± 8.89 | 52.38 ± 8.68 | 273.20 | <0.001 | a(<0.001)  b(<0.001)  c(<0.001) |

**Note:** Post hoc comparisons were corrected with Bonferroni–Holm method; a, ADHD_ODD_- versus HC; b, ADHD_ODD-_ versus ADHD_ODD+_; c, HC versus ADHD_ODD+_

**Abbreviation:** ADHD = Attention deficit hyperactivity disorder; ADHD_ODD-_ = ADHD children, none of the ODD item of NICHQ Vanderbilt ADHD screen were greater or equal to 2; ADHD_ODD+_= ADHD children, at least 2 ODD items of NICHQ Vanderbilt ADHD screen were greater or equal to 2; HC = healthy control; IQ = Estimated intelligence quotient; IA/H/C= Inattentive/Hyperactive-impulsive/Combined subtype.

**Table S7. Comparison of FC in ADHD_ODD-_, ADHD_ODD+_ and cHC**

| **Dependent variables** | **ADHD_ODD-_**  **(mean ± SD)** | **cHC**  **(mean ± SD)** | **ADHD_ODD+_**  **(mean ± SD)** | ***F*** | **Adjusted *p*** | **Post hoc comparisons**  **(*p* value)** |
| --- | --- | --- | --- | --- | --- | --- |
| **DAN(L)-DAN(L)** | 0.4187 ± 0.3064 | 0.5433 ± 0.2035 | 0.4285 ± 0.2572 | 1.418 | 0.246 | / |
| **DAN(L)-DMN(R)** | 0.2817 ± 0.1530 | 0.3923 ± 0.1350 | 0.2992 ± 0.1659 | 4.750 | 0.0125 | a(0.014) |
| **DAN(L)-DAN(R)** | 0.3002 ± 0.1919 | 0.4650 ± 0.1663 | 0.3417 ± 0.2008 | 7.349 | 0.0017 | a(0.001)  c(0.020) |
| **VN(L)-SMN(R)** | 0.2385 ± 0.1688 | 0.3952 ± 0.2020 | 0.2738 ± 0.1851 | 7.143 | 0.0017 | a(0.001)  c(0.017) |
| **VN(L)-DAN(R)** | 0.2969 ± 0.2205 | 0.4890 ± 0.1983 | 0.3225 ± 0.2446 | 9.057 | 0.001 | a(<0.001)  c(0.002) |

**Note:** After adjusting for age, sex, and IQ; Post hoc comparisons were corrected with Bonferroni–Holm method; a, ADHD_ODD_- versus HC; b , ADHD_ODD-_ versus ADHD_ODD+_; c, HC versus ADHD_ODD+._

**Abbreviation:** ADHD = Attention deficit hyperactivity disorder; ADHD_ODD-_ = ADHD children, none of the ODD item of NICHQ Vanderbilt ADHD screen were greater or equal to 2; ADHD_ODD+_= ADHD children, at least 2 ODD items of NICHQ Vanderbilt ADHD screen were greater or equal to 2; HC = healthy control; VN = visual network; SMN = somatomotor network; DAN = dorsal attention network; VAN = ventral attention network; DMN = default mode network; L = left; R = right.

**Table S8. Correlation between FC and CD, ODD symptoms in children with ADHD after controlling other comorbidities** **and core symptoms.**

| **FC** | **CD** | | | |  | **ODD** | | | |
| --- | --- | --- | --- | --- | --- | --- | --- | --- | --- |
|  | ***r*^*^** | ***P_FDR_*^*^** | ***r*^#^** | ***P_FDR_*^#^** |  | ***r*^*^** | ***P_FDR_*^*^** | ***r*^#^** | ***P_FDR_*^#^** |
| **DAN(L)-DAN(L)** | 0.165 | 0.040 | 0.162 | 0.040 |  | -0.018 | 0.411 | -0.017 | 0.418 |
| **DAN(L)-DMN(R)** | 0.222 | 0.015 | 0.217 | 0.020 |  | 0.052 | 0.339 | 0.057 | 0.328 |
| **DAN(L)-DAN(R)** | 0.159 | 0.040 | 0.160 | 0.040 |  | 0.095 | 0.339 | 0.096 | 0.328 |
| **VN(L)-SMN(R)** | 0.139 | 0.053 | 0.135 | 0.060 |  | 0.081 | 0.339 | 0.084 | 0.328 |
| **VN(L)-DAN(R)** | 0.126 | 0.060 | 0.123 | 0.065 |  | -0.049 | 0.339 | 0.051 | 0.328 |

**Note: ***Covariate-adjusted Spearman’s Rank Correlation, one-tailed, after adjusting for age, sex, IQ and comorbidities; FDR corrected.

^#^Covariate-adjusted Spearman’s Rank Correlation, one-tailed, after adjusting for age, sex, IQ and comorbidities and core symptoms; FDR corrected.

**Abbreviation:** VN = visual network; SMN = somatomotor network; DAN = dorsal attention network; VAN = ventral attention network; DMN = default mode network; L = left; R = right.

**Table S9. Comparison of FC in ADHD_CD-_, ADHD_CD+_ and cHC after controlling other comorbidities**

| **Dependent variables** | **ADHD_CD-_** | **cHC** | **Subthreshold ADHD_CD+_** | ***F*** | **Adjusted *P*** | **Post hoc comparisons**  **(*p* value)** |
| --- | --- | --- | --- | --- | --- | --- |
| **DAN(L)-DAN(L)** | 0.323 | 0.592 | 0.5495 | 1.347 | 0.290 | - |
| **DAN(L)-DMN(R)** | 0.168 | 0.386 | 0.461 | 10.975 | 0.0025 | b(0.001) |
| **DAN(L)-DAN(R)** | 0.192 | 0.507 | 0.514 | 6.846 | 0.00133 | b(0.011) |
| **VN(L)-SMN(R)** | 0.107 | 0.416 | 0.394 | 10.714 | 0.0025 | a(0.042)  b(0.002) |
| **VN(L)-DAN(R)** | 0.137 | 0.544 | 0.488 | 6.138 | 0.0137 | b(0.020) |

**Note:** After adjusting for age, sex, and IQ; Post hoc comparisons were corrected with Bonferroni–Holm method; a, ADHD_CD-_ versus HC; b, ADHD_CD-_ versus ADHD_CD+_; c, HC versuss ADHD_CD+._

**Abbreviation:** ADHD = Attention deficit hyperactivity disorder; ADHD_CD_- = ADHD children, none of the CD item of NICHQ Vanderbilt ADHD screen were greater or equal to 2; ADHD_CD+_ = ADHD children, at least 2 CD items of NICHQ Vanderbilt ADHD screen were greater or equal to 2；HC = healthy control; VN = visual network; SMN = somatomotor network; DAN = dorsal attention network; VAN = ventral attention network; DMN = default mode network; L = left; R = right.

**Table S10. Comparison of FC in ADHD_CD-_, ADHD_CD+_ and cHC after controlling other comorbidities and core symptoms**

| **Dependent variables** | **ADHD_CD-_** | **cHC** | **Subthreshold ADHD_CD+_** | ***F*** | **Adjusted *P*** | **Post hoc comparisons**  **(*p* value)** |
| --- | --- | --- | --- | --- | --- | --- |
| **DAN(L)-DAN(L)** | 0.385 | 0.383 | 0.642 | 1.597 | 0.237 | - |
| **DAN(L)-DMN(R)** | 0.177 | 0.356 | 0.482 | 10.268 | 0.005 | b(0.004) |
| **DAN(L)-DAN(R)** | 0.226 | 0.393 | 0.594 | 7.105 | 0.0117 | b(0.012) |
| **VN(L)-SMN(R)** | 0.119 | 0.376 | 0.423 | 10.193 | 0.005 | b(0.006) |
| **VN(L)-DAN(R)** | 0.183 | 0.391 | 0.595 | 6.413 | 0.0137 | b(0.017) |

**Note:** After adjusting for age, sex,IQ and other comorbidities and core symptoms; Post hoc comparisons were corrected with Bonferroni–Holm method; a, ADHD_CD-_ versus HC; b, ADHD_CD-_ versus ADHD_CD+_; c, HC versus ADHD_CD+._

**Abbreviation:** ADHD = Attention deficit hyperactivity disorder; ADHD_CD_- = ADHD children, none of the CD item of NICHQ Vanderbilt ADHD screen were greater or equal to 2; ADHD_CD+_ = ADHD children, at least 2 CD items of NICHQ Vanderbilt ADHD screen were greater or equal to 2；HC = healthy control; VN = visual network; SMN = somatomotor network; DAN = dorsal attention network; VAN = ventral attention network; DMN = default mode network; L = left; R = right.

**Table S11. Comparison of FC in ADHD_ODD-_, ADHD_ODD+_ and cHC after controlling other comorbidities**

| **Dependent variables** | **ADHD_ODD-_** | **cHC** | **ADHD_ODD+_** | ***F*** | **Adjusted *P*** | **Post hoc comparisons**  **(*p* value)** |
| --- | --- | --- | --- | --- | --- | --- |
| **DAN(L)-DAN(L)** | 0.435 | 0.521 | 0.4335 | 1.556 | 0.215 | - |
| **DAN(L)-DMN(R)** | 0.289 | 0.383 | 0.301 | 4.624 | 0.0125 | a(0.013)  c(0.046) |
| **DAN(L)-DAN(R)** | 0.307 | 0.462 | 0.338 | 8.583 | 0.0015 | a(<0.001)  c(0.006) |
| **VN(L)-SMN(R)** | .244 | 0.387 | 0.276 | 6.408 | 0.0033 | a(0.002)  c(0.025) |
| **VN(L)-DAN(R)** | 0.306 | 0.477 | 0.326 | 7.541 | 0.0025 | a(0.001)  c(0.005) |

**Note:** After adjusting for age, sex, IQ and other comorbidities; Post hoc comparisons were corrected with Bonferroni–Holm method;

a, ADHD_ODD_- versus HC; b, ADHD_ODD-_ versus ADHD_ODD+_; c, HC versus ADHD_ODD+._

**Abbreviation:** ADHD = Attention deficit hyperactivity disorder; ADHD_ODD-_ = ADHD children, none of the ODD item of NICHQ Vanderbilt ADHD screen were greater or equal to 2; ADHD_ODD+_= ADHD children, at least 2 ODD items of NICHQ Vanderbilt ADHD screen were greater or equal to 2；HC = healthy control; VN = visual network; SMN = somatomotor network; DAN = dorsal attention network; VAN = ventral attention network; DMN = default mode network; L = left; R = right.

**Table S12. Comparison of FC in ADHD_ODD-_, ADHD_ODD+_ and cHC after controlling other comorbidities and core symptoms**

| **Dependent variables** | **ADHD_ODD-_** | **cHC** | **ADHD_ODD+_** | ***F*** | **Adjusted *P*** | **Post hoc comparisons**  **(*p* value)** |
| --- | --- | --- | --- | --- | --- | --- |
| **DAN(L)-DAN(L)** | 0.417 | 0.577 | 0.400 | 1.490 | 0.229 | - |
| **DAN(L)-DMN(R)** | 0.285 | 0.399 | 0.293 | 2.161 | 0.149 | - |
| **DAN(L)-DAN(R)** | 0.299 | 0.490 | 0.323 | 4.471 | 0.065 | a (0.013) |
| **VN(L)-SMN(R)** | 0.236 | 0.418 | 0.260 | 3.538 | 0.080 | a(0.034) |
| **VN(L)-DAN(R)** | 0.302 | 0.495 | 0.318 | 3.029 | 0.087 | - |

**Note:** After adjusting for age, sex, IQ , other comorbidities and core symptoms; Post hoc comparisons were corrected with Bonferroni–Holm method; a, ADHD_ODD_- versus HC; b, ADHD_ODD-_ versus ADHD_ODD+_; c, HC versus ADHD_ODD+_

**Abbreviation:** ADHD = Attention deficit hyperactivity disorder; ADHD_ODD-_ = ADHD children, none of the ODD item of NICHQ Vanderbilt ADHD screen were greater or equal to 2; ADHD_ODD+_= ADHD children, at least 2 ODD items of NICHQ Vanderbilt ADHD screen were greater or equal to 2；HC = healthy control; VN = visual network; SMN = somatomotor network; DAN = dorsal attention network; VAN = ventral attention network; DMN = default mode network; L = left; R = right.

**Appendix 2. FCs differences of** **ADHD_CD+_/ADHD_ODD+_, ADHD_CD-_/ADHD_ODD-_ and cHC**

To render the results readily interpretable, we compared the FCs differences among ADHD_CD-_, subthreshold ADHD_CD+_ and HC, and among ADHD_ODD_-, ADHD_ODD+_ and HC, respectively.

We first counted the number of items rated above 2 (N) under the DBD dimension for each child. Two groups were selected from 172 children with ADHD according to scores of NICHQ Vanderbilt ADHD screen for categorical analyses. One group (ADHD_CD+_/ADHD_ODD+_) is the patients who meet the criterion of CD or ODD according to NICHQ Vanderbilt (ADHD_CD+_: N in CD domain ≥ 3; ADHD_ODD+_: N in ODD domain ≥ 4). The other group (ADHD_CD-_/ADHD_ODD-_) is none item of the CD or/and ODD dimension beyond two (ADHD_CD-_: N in CD domain = 0 regardless of ODD; ADHD_ODD-_: N in ODD domain = 0 without CD). The ADHD_CD+_ and ADHD_CD-_ , ADHD_ODD+_ and ADHD_ODD-_ were 1:1 age‐ and sex‐matched, respectively. Considering the relatively low incidence rate of ADHD_CD+_. Thus, we redefined “subthreshold ADHD_CD+_” as “N in CD domain ≥ 2” in the current study to enhance statistic power. Then, we randomly selected one group of cHC that were 1:1 sex and age- matched with cADHD using R. Finally, we compared HC to the previously mentioned ADHD_CD+_/ADHD_ODD+_ and ADHD_CD-_/ADHD_ODD-_ groups.
